# Preparative Separation of Antioxidants from Sea Buckthorn and Its Antioxidant Activity In Vitro via Endothelial Function Regulation

**DOI:** 10.3390/ijms27093757

**Published:** 2026-04-23

**Authors:** Yurong Cheng, Wenjuan Kang, Jingwen Hu, Xueru Fan, Xingmei Nan, Zonghao Zhang, Fang Yang

**Affiliations:** 1 School of Pharmacy, Qinghai University, Xining 810016, China; cyr225118@163.com (Y.C.);; 2 College of Animal Husbandry and Veterinary Science, Qinghai University, Xining 810016, China

**Keywords:** sea buckthorn, preparative separation, vitro analysis, antioxidant activity, molecular docking

## Abstract

Sea buckthorn, a homologue of medicine and food, contains a host of bioactives that can prevent many diseases, especially cardiovascular diseases. The association between oxidative stress (OS) and cardiovascular diseases (CVDs) has been well-established, with OS ultimately leading to CVDs through lipid peroxidation and other mechanisms. In this study, antioxidant components were isolated from sea buckthorn by polyamide medium-pressure chromatography coupled with an HPLC-DPPH activity screening system. Two potential compounds were isolated and identified as Tetrahydroharmol and Isorhamnetin3-O-(6-O-E-sinapoyl)-β-D-glucopyranosyl-(1-2)-β-D-glucopyranoside-7-O-α-L-rhamnopyranoside. Molecular docking technology was used to explore the binding ability of two antioxidant active components to target proteins (LDH, SOD, Nrf2, iNOS, and eNOS). In addition, the antioxidant capacity was determined by EA.hy926 human umbilical vein endothelial fusion cell experiments. The results demonstrate the efficacy of this method for isolating high-purity antioxidants from sea buckthorn. These two activity compounds exhibit potential effects against cardiovascular diseases through antioxidant mechanisms.

## 1. Introduction

Sea buckthorn (*Hippophae rhamnoides* Linn. subsp. Sinensis Rousi) is an important medicinal and edible plant with a long history of use in Tibetan traditional medicine. Its antioxidant, anti-inflammatory, and cardiovascular protective effects have garnered significant attention [1,2]. The flavonoids and sterols in sea buckthorn possess lipid-lowering and vasoregulatory functions, while its phenolic compounds and vitamin C exhibit antioxidant properties [3,4,5]. However, current research on sea buckthorn’s antioxidant activity remains largely confined to the extraction stage, with limited studies on individual antioxidant compounds. Existing extraction and purification methods suffer from low efficiency and high costs. Therefore, it is necessary to develop a new method for extracting and separating antioxidant monomer compounds from sea buckthorn.

The occurrence of cardiovascular disease is closely related to oxidative stress [6]. Under physiological conditions, free radicals play crucial roles as signaling molecules; however, their excessive production leads to oxidative stress, triggering a series of pathological processes, including endothelial cell membrane damage, nitric oxide (NO) dysfunction, inflammatory activation, and apoptosis, ultimately resulting in vascular dysfunction and cardiovascular diseases [7]. Vascular endothelium is central to maintaining vascular homeostasis. Oxidative stress-induced endothelial injury may play a key role in the development of diseases such as atherosclerosis, heart failure, hypertension and myocardial hypertrophy [8,9,10]. Antioxidants can neutralize free radicals, repair damage and regulate signaling pathways to maintain cell homeostasis. The pleiotropic effects of antioxidants, as well as the anti-inflammatory properties of some well-known antihypertensive drugs and statins, to some extent support the use of antioxidants in vascular diseases. According to different sources, antioxidants can be divided into endogenous and exogenous antioxidants. Exogenous antioxidants are of two categories: natural and synthetic [11,12]. However, some synthetic antioxidants exhibit high toxicity, whereas natural antioxidants possess minimal toxicity and side effects. Beyond scavenging excess free radicals in the body, they have positive significance for the prevention and treatment of related diseases [13]. Therefore, identifying natural antioxidants is of significant importance.

Traditional separation methods have the drawback of loss of antioxidant active ingredients. In order to promote the sustainable development and utilization of medicinal plant resources, modern separation and purification technology is urgently needed [14]. Medium-Pressure Liquid Chromatography (MPLC) has been widely used in the pretreatment and large-scale separation of natural products due to its advantages of high efficiency, visualization and large sample loading [15]. In this study, MPLC with a polyamide filler was used to pretreat sea buckthorn extract to achieve effective separation of different components and avoid the pollution of a subsequent high-performance liquid chromatography (HPLC) stationary phase. High pressure liquid chromatography has been extensively employed in the separation and purification of natural product components since it can achieve efficient and targeted separation in the face of complex samples [16]. In recent years, online HPLC-DPPH analysis technology has been successfully developed and applied to the rapid screening and structural identification of antioxidants in plant crude extracts. Based on DPPH free radical scavenging activity, online HPLC-DPPH analysis technology was used to quickly and efficiently screen and identify the antioxidant active components in the crude extract of sea buckthorn [17]. Direct validation of the biological activity of numerous isolated monomers is costly and time-consuming [18]. Molecular docking simulation technology provides an efficient preliminary screening approach for this purpose. This technology can be utilized to predict the binding mode and affinity of potential antioxidant monomers (ligands) and key oxidative stress regulatory targets (receptors), theoretically evaluating their potential to interfere with oxidative pathways.

Therefore, the aim of this study is to establish an efficient method for the rapid isolation of high-purity antioxidant monomers from sea buckthorn using polyamide medium-pressure chromatography combined with an online HPLC-DPPH screening system and to preliminarily evaluate their antioxidant activities in vitro.

## 2. Results

### 2.1. Medium-Pressure Chromatography for Sea Buckthorn Sample Pretreatment

**Figure 1:**
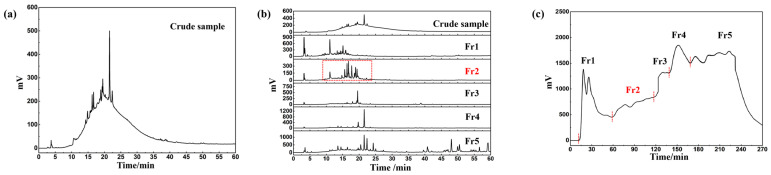
Analytical chromatogram of sea buckthorn extract (**a**). The analytical chromatogram of sea buckthorn and Fr1 to Fr5 (**b**). The polyamide separation chromatogram of sea buckthorn (**c**).

Sea buckthorn pomace (4.0 kg) was extracted with 70% methanol and mixed with polyamide (120.0 g) for drying. Finally, 120.0 g of extract (yield: 3.0%) was obtained. The sea buckthorn extract was analyzed by chromatography, and the analysis chromatogram is shown in Figure 1a. According to the analysis chromatogram, the peaks in the chromatogram show an irregular phenomenon, and the absorption value of the peak does not start from 0. The preliminary analysis showed that this phenomenon may stem from the large amount of polymer contained in the extract. The chromatographic analysis of the crude sample and each fraction after pretreatment with polyamide MPLC are shown in Figure 1b. It can be seen that there is no significant cross between the components. These five components contain most of the chromatographic peaks in the crude extract. The crude extract of 60.0 g of sea buckthorn fruit was pretreated by polyamide medium-pressure chromatography. The chromatogram of the sample analysis is shown in Figure 1c.

**Figure 2:**
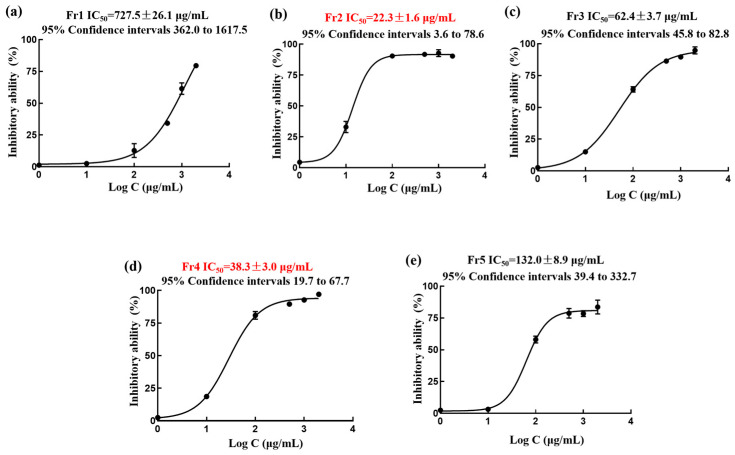
DPPH inhibition activities of Fr1, Fr2, Fr3, Fr4, and Fr5 at different concentrations were fitted with a logistic function to count the IC_50_ value (**a**–**e**).

Finally, five fractions were recovered, with a recovery rate of 48.3%, and the weights were Fr1 (10.58 g), Fr2 (2.85 g), Fr3 (0.93 g), Fr4 (2.28 g) and Fr5 (12.33 g), respectively. The results of DPPH scavenging experiments of five fractions of sea buckthorn are shown in Figure 2a–e. The half inhibitory concentrations (IC_50_) of the five fractions were calculated to be Fr1 (727.5 ± 26.1 μg mL^−1^), Fr2 (22.3 ± 1.6 μg mL^−1^), Fr3 (62.4 ± 3.7 μg mL^−1^), Fr4 (38.3 ± 3.0 μg mL^−1^) and Fr5 (132.0 ± 8.9 μg mL^−1^). According to the screening results of the five components, Fr2 has significant antioxidant activity.

### 2.2. Antioxidant Activity and Cell Viability

**Figure 3:**
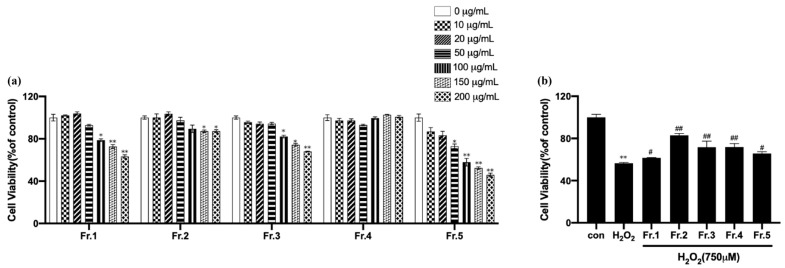
The cell viability of Fr1, Fr2, Fr3, Fr4, and Fr5 (**a**). * *p* < 0.05, ** *p* < 0.01 vs. 0 μg mL^−1^. Effects of Fr1 to Fr5 on the viability of EA.hy926 cells injured by H_2_O_2_ (**b**). Compared with the control group, ** *p* < 0.01; compared with the H_2_O_2_ group, ^#^ *p* < 0.05, and ^##^ *p* < 0.01.

The effects of the five components on the viability of EA.hy926 cells were detected by MTT assay. As shown in Figure 3a, the five components had no effect on the viability of EA.hy926 cells when less than or equal to 20 μg mL^−1^, and the relative survival rate of EA.hy926 cells clustered above 80%. At concentrations ≥ 100 μg mL^−1^, cell viability significantly decreased (*p* < 0.05), with Fr3 and Fr5 exhibiting a pronounced concentration-dependent decrease in survival rates. When concentrations exceeded 50 μg mL^−1^, Fr3 and Fr5 demonstrated notable cytotoxicity, while the other components showed weaker toxicity. Therefore, 20 μg mL^−1^ was selected as the treatment concentration for subsequent experiments.

After establishing the oxidative damage model induced by H_2_O_2_, the five components were added and incubated with cells at 20 μg mL^−1^ to investigate their effects on EA.hy926 cell viability. The groups were divided into a blank group (Con), a model group (H_2_O_2_), and drug groups (H_2_O_2_ + different fractions of sea buckthorn). Compared with a control group, cell viability was significantly decreased after adding H_2_O_2_ (*p* < 0.01). Compared with the H_2_O_2_ group, all five components could increase cell viability (*p* < 0.05), and Fr2 has the most significant effect in Figure 3b. Hence, Fr2 was selected for the next experiment.

### 2.3. Isolation and Identification of Fr2 Antioxidant

**Figure 4:**
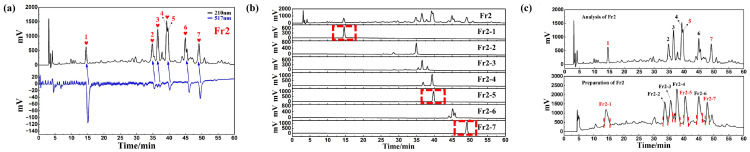
The activity screening chromatogram of Fr2 (**a**). The analytical chromatogram of Fr2 and Fr2-1 to Fr2-7 (**b**). Comparison of analysis chromatogram and preparation chromatogram of Fr2 (**c**).

To obtain good separation results, the chromatographic conditions for Fr2 were optimized using a ReproSil-Pur C18 AQ analytical column, and the active peaks in Fr2 were qualitatively identified by an online HPLC-DPPH activity screening system. Finally, seven obvious active peaks were identified from Fr2. The chromatogram of activity screening is presented in Figure 4a. By comparing the analytical chromatogram to the preparative chromatogram, it was observed that the peaks in the preparative chromatogram shifted forward compared to those in the analytical chromatogram; however, this did not affect the separation and preparation of the free radical inhibitor. Seven fractions of Fr2-1 (143.13 mg), Fr2-2 (107.34 mg), Fr2-3 (139.41 mg), Fr2-4 (162.99 mg), Fr2-5 (129.63 mg), Fr2-6 (165.09 mg), and Fr2-7 (97.50 mg) were obtained by preparative high-pressure liquid chromatography (HPLC) after 19 repeated separations and preparations of seven fractions of Fr2 (97.50 mg), as shown in Figure 4b. Based on the activity-screening results, the active peaks of Fr2 were separated according to their activity-oriented targets. The analytical and preparative chromatogram of Fr2 is presented in Figure 4c.

**Figure 5:**
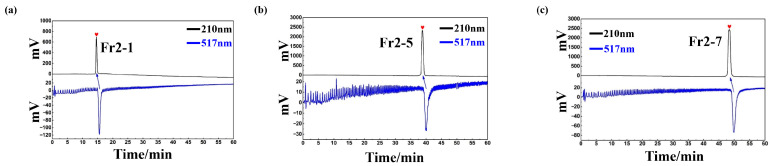
Activity screening chromatograms of Fr2-1, Fr2-5 and Fr2-7 (**a**–**c**).

The results of the chromatograms reveal that there was no crossover between the components and that there was only a single main peak in the Fr2-1, Fr2-5 and Fr2-7 components with high purity. Thus, the antioxidant activity of Fr2-1, Fr2-5 and Fr2-7 was verified using an on-line HPLC-DPPH activity screening system, and the chromatograms of Fr2-1, Fr2-5 and Fr2-7 are presented in Figure 5a–c.

**Figure 6:**
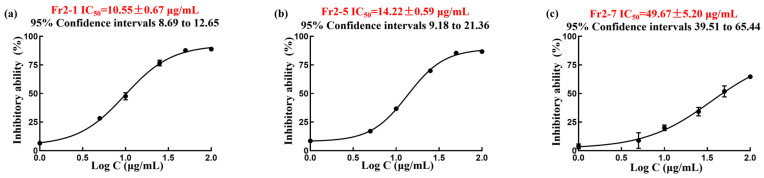
DPPH scavenging of Fr2-1, Fr2-5 and Fr2-7 (**a**–**c**).

The DPPH scavenging experiments of Fr2-1, Fr2-5 and Fr2-7 are presented in Figure 6a–c, and the half-inhibitory concentrations (IC_50_) of the Fr2-1, Fr2-5 and Fr2-7 components were calculated as to be 10.55 ± 0.67 μg mL^−1^, 14.22 ± 0.59 μg mL^−1^ and 49.67 ± 5.20 μg mL^−1^. Meanwhile, on the basis of physical and chemical properties and NMR spectroscopic data, the structures of the monomer compounds Fr2-1, Fr2-5 and Fr2-7 were identified (Table 1).

**Figure 7:**
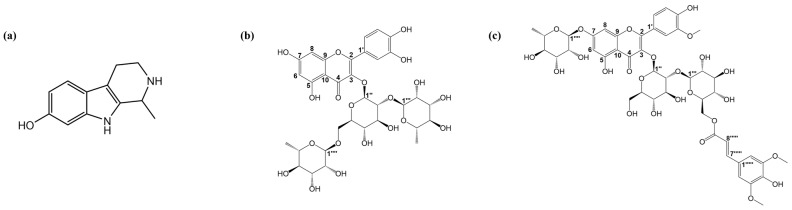
Structure of compounds Fr2-1 (**a**), Fr2-5 (**b**) and Fr2-7 (**c**).

The compound Fr2-1 (143.13 mg) appeared as a white powder with the following characteristics (Figure 7a): ESI-MS *m*/*z* 201.11, [M-H]-, molecular formula C_12_H_14_N_2_O; ^1^H-NMR (MeOH-*d_4_* 600 MHz): *δ*_H_ 7.25 (1H, d, *J* = 8.5 Hz, H-5), 6.75 (1H, d, *J* = 1.9 Hz, H-8), 6.61 (1H, dd, *J* = 8.5, 1.9 Hz, H-6), 4.69 (1H, q, *J* = 6.7 Hz, H-1), 3.67 (1H, m, H-3a), 3.41 (1H, m, H-3b), 3.03 (1H, m, H-4a), 2.96 (1H, m, H-4b), 1.68 (3H, d, *J* = 6.7 Hz, H-10); ^13^C-NMR (MeOH-*d*_4_, 150 MHz): *δ*C 155.0 (s, C-7), 139.4 (s, C-8a), 129.3 (s, C-9a), 121.3 (s, C-4b), 119.6 (d, C-5), 110.7 (d, C-6), 106.7 (s, C-4a), 97.8 (d, C-8), 50.7 (d, C-1), 42.6 (t, C-3), 19.6 (t, C-4), 17.8 (q, C-10). See Supplementary Materials Fr2-1. The results are consistent with those reported in the literature, and the final identification of the compound Fr2-1 characterized it as Tetrahydroharmol [19].

The compound Fr2-5 (129.60mg) showed as a yellow powder possessing the following characteristics: ESI-MS *m*/*z*: 755.55, [M-H]-, molecular formula C_33_H_40_O_20_; ^1^H-NMR (MeOH-*d*_4_, 600 MHz): *δ*H 7.60 (1H, dd, *J* = 8.4, 2.1 Hz, H-6′), 7.58 (1H, d, *J* = 2.1 Hz, H-2′), 6.86 (1H, d, *J* = 8.4 Hz, H-5′), 6.37 (1H, d, *J* = 2.1 Hz, H-8), 6.18 (1H, d, *J* = 2.1 Hz, H-6); 3-glc: 5.59 (1H, d, *J* = 7.7 Hz, H-1″), 3.81 (1H, dd, *J* = 11.3, 1.5 Hz, H-6″a); 2″-rha: 5.21 (1H, d, *J* = 1.6 Hz, H-1‴), 3.99 (1H, dd, *J* = 3.3, 1.6 Hz, H-2‴), 3.78 (1H, dd, *J* = 9.6, 3.3 Hz, H-3‴), 4.07 (1H, m, H-5‴), 0.99 (3H, d, *J* = 6.2 Hz, H-6‴); 6″-rha: 4.50 (1H, d, *J* = 1.6 Hz, H-1′′′′), 3.57 (1H, dd, *J* = 3.4, 1.6 Hz, H-2′′′′), 3.48 (1H, dd, *J* = 9.5, 3.4 Hz, H-3′′′′), 3.41 (1H, m, H-5′′′′), 1.07 (3H, d, *J* = 6.2 Hz, H-6′′′′); ^13^C-NMR (MeOH-*d*_4_, 150 MHz): *δ*C 179.3 (C-4), 165.6 (C-7), 163.2 (C-5), 158.9 (C-9), 158.5 (C-2), 149.6 (C-4′), 145.9 (C-3′), 134.5 (C-3), 123.5 (C-1′), 123.5 (C-6′), 117.4 (C-5′), 116.0 (C-2′), 105.9 (C-10), 99.7 (C-6), 94.7 (C-8); 3-glc: 100.5 (C-1″), 80.0 (C-2″), 78.9 (C-3″), 71.9 (C-4″), 77.1 (C-5″), 68.3 (C-6″); 2″-rha: 102.7 (C-1‴), 72.3 (C-2‴), 72.4 (C-3‴), 74.1 (C-4‴), 70.0 (C-5‴), 17.5 (C-6‴); 6″-rha: 102.3 (C-1′′′′), 72.1 (C-2′′′′), 72.3 (C-3′′′′), 73.9 (C-4′′′′), 69.7 (C-5′′′′), 17.8 (C-6′′′′). The results are consistent with those reported in the literature, and the final identification of the compound Fr2-5 characterized it as Manghaslin [20] (Figure 7b).

As can be seen in Figure 7c, the compound Fr2-7 (97.5 mg) appeared as a yellow powder possessing the following characteristics: ESI-MS *m/z* 991.39, [M-H]-, molecular formula C_45_H_52_O_25_; ^1^H-NMR (MeOH-*d*_4_, 600 MHz): *δ*H 7.68 (1H, dd, *J* = 8.6, 2.1 Hz, H-6′), 7.68 (1H, brs, H-2′), 6.91 (1H, d, *J* = 8.6 Hz, H-5′), 6.37 (1H, d, *J* = 2.1 Hz, H-8), 6.29 (1H, d, *J* = 2.1 Hz, H-6), 3.96 (3H, s, OCH_3_); 3-glc: 5.29 (1H, d, *J* = 7.8 Hz, H-1″); 2″-glc: 4.67 (1H, d, *J* = 7.7 Hz, H-1‴), 4.44 (1H, dd, *J* = 11.7, 7.1 Hz, H-6‴a), 4.31 (1H, dd, *J* = 11.7, 2.3 Hz, H-6‴b); 7-rha: 5.50 (1H, d, *J* = 1.7 Hz, H-1′′′′), 4.07 (1H, dd, *J* = 3.5, 1.7 Hz, H-2′′′′), 3.81 (1H, dd, *J* = 9.4, 3.5 Hz, H-3′′′′), 1.22 (3H, d, *J* = 6.1 Hz, H-6′′′′); O-Sin: 6.46 (2H, s, H-2′′′′′, H-6′′′′′), 7.25 (1H, d, *J* = 15.9 Hz, H-7′′′′′), 6.02 (1H, d, *J* = 15.9 Hz, H-8′′′′′), 3.75 (6H, s, OCH3); ^13^C-NMR (MeOH-*d*_4_, 150 MHz): *δ*C 179.9 (s, C-4), 163.3 (s, C-7), 162.6 (s, C-5), 158.5 (s, C-2), 157.6 (s, C-9), 151.1 (s, C-4′), 148.7 (s, C-3′), 135.3 (s, C-3), 125.1 (d, C-6′), 122.9 (s, C-1′), 116.3 (d, C-5′), 114.0 (d, C-2′), 107.3 (s, C-10), 100.2 (d, C-6), 95.3 (d, C-8), 57.1 (q, OCH3); 3-glc: 100.4 (d, C-1″), 85.2 (d, C-2″), 77.7 (d, C-3″), 71.2 (d, C-4″), 78.4 (d, C-5″), 62.3 (t, C-6″); 2″-glc: 106.4 (d, C-1‴), 75.6 (d, C-2‴), 77.8 (d, C-3‴), 72.1 (d, C-4‴), 76.3 (d, C-5‴), 64.6 (t, C-6‴); 7-rha: 99.8 (d, C-1′′′′), 71.7 (d, C-2′′′′), 72.1 (d, C-3′′′′), 73.7 (d, C-4′′′′), 71.1 (d, C-5′′′′), 18.1 (q, C-6′′′′); O-Sin: 126.2 (s, C-1′′′′′), 106.2 (d, C-2′′′′′, C-6′′′′′), 149.0 (s, C-3′′′′′, C-5′′′′′), 139.1 (s, C-4′′′′′), 146.7 (d, C-7′′′′′), 115.3 (d, C-8′′′′′), 168.7 (s, C-9′′′′′), 56.5 (q, OCH_3_). See Supplementary Materials Fr2-7. The results are consistent with those reported in the literature, and the final identification of the compound Fr2-7 characterized it as Isorhamnetin 3-O-(6-O-E-sinapoyl)-D-glucopyranosyl-(1-2)-β-D-glucopyranoside7-O-α-L-rhamnopyranoside [21].

Through the preliminary online DPPH-HPLC screening system and cells, it was found that Fr2-1, Fr2-5 and Fr2-7 had relatively good antioxidant activities. However, due to the limited amount of purified Fr2-5 available after preparative isolation, only Fr2-1 and Fr2-7 were included in the subsequent cell-based assays. The other four fractions, namely Fr2-2, Fr2-3, Fr2-4, and Fr2-6, are not pure compounds and need to separate further. Therefore, the vitro activity test focused on the antioxidant activities of these two compounds. Moreover, the reliability of the online HPLC-DPPH activity screening system was verified.

### 2.4. Molecular Docking Results

**Figure 8:**
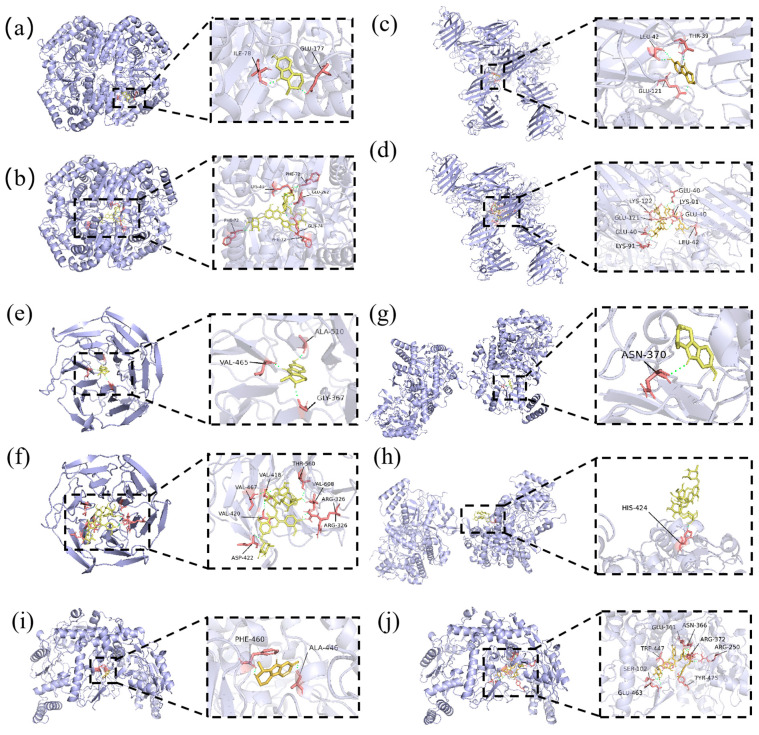
A three-dimensional interaction diagram of LDH with Fr2-1 (**a**) and Fr2-7 (**b**). A three-dimensional interaction diagram of SOD with Fr2-1 (**c**) and Fr2-7 (**d**). A three-dimensional interaction diagram of Nrf2 with Fr2-1 (**e**) and Fr2-7 (**f**). A three-dimensional interaction diagram of iNOS with Fr2-1 (**g**) and Fr2-7 (**h**). A three-dimensional interaction diagram of eNOS with Fr2-1 (**i**) and Fr2-7 (**j**).

Through molecular docking simulations, the ligand was placed near the target active site. Algorithms searched for the optimal orientation and location of ligands to achieve the lowest-energy conformation and calculate binding energy. A binding energy < −5 kcal mol^−1^ indicates spontaneous binding. The binding ability of the compounds Fr2-1 and Fr2-7 with cardiovascular disease-related target proteins (LDH, SOD, Nrf2, iNOS, and eNOS) was evaluated, and the stability and affinity of the binding were predicted [22,23,24,25,26]. Detailed docking information is shown in Table 2. It is clear that the two antioxidant monomer compounds showed strong binding energy with the three proteins. Specifically, Fr2-1 binds to LDH through Glu-177 and Ile-78 (Figure 8a), and Fr2-7 binds to LDH through the active structure composed of Phe-7, Lys-43 and Glu-262 (Figure 8b). Fr2-1 binds to SOD through THR-39, LEU-42, and GLU-121 (Figure 8c), and Fr2-7 binds to SOD through LEU-42, GLU-40, LYS-91, LYS-122, and GLU-121 (Figure 8d). Fr2-1 binds to Nrf2 through Val-465, Ala-510, and Gly-367 hydrogen bonds (Figure 8e). Fr2-7 interacts with Nrf2 by forming hydrogen bonds through Asp-422, Val-420, Val-467, Val-418, Thr-560, Val-608, and Arg-326 amino acid residues (Figure 8f). Fr2-1 binds to iNOS through Asn-370 (Figure 8g), and Fr2-7 interacts with iNOS through His-424 (Figure 8h). Fr2-1 binds to eNOS through ALA-446 and PHE-460 (Figure 8i), and Fr2-7 binds to eNOS through SER-102, ARG-250, GLU-361, ASN-366, ARG-372, TRP-447, GLU-463 and TYR-475 hydrogen bonds (Figure 8j). This indicates that there is a specific interaction mode between Fr2-1 and Fr2-7 and these five proteins. This is of great significance for the subsequent study of their antioxidant activity in vitro.

### 2.5. Fr2 In Vitro Antioxidant Test Results

#### 2.5.1. Determination of Endothelial Cell Viability

**Figure 9:**
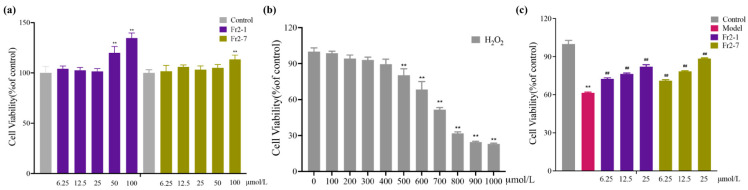
The influence of Fr2-1 and Fr2-7 on the viability of EA.hy926 endothelial cells (**a**). Influence of H_2_O_2_ concentration on the viability of EA.hy926 endothelial cells (**b**). MTT assay was used to assess the effects of a range of Fr2-1 and Fr2-7 concentrations on the viability of H_2_O_2_-injured EA.hy926 endothelial cells (**c**). Compared with the control group, ** *p* < 0.01; compared with the model group, ^##^ *p* < 0.01.

After the application of sea buckthorn compounds at various concentrations to EA.hy926 endothelial cells for 24 h, Fr2-1 enhanced the viability of the EA.hy926 endothelial cells to different degrees at 6.25~100 μmol L^−1^ (*p* < 0.05). Fr2-7 enhanced the viability of endothelial cells at concentrations other than 6.25 μmol L^−1^ (*p* < 0.05) but did not exhibit a dose dependence. The relative viability of the EA.hy926 endothelial cells was increased by Fr2-1 and Fr2-7 at concentrations above 6.25 μmol L^−1^ (*p* < 0.05) (Figure 9a). Therefore, 6.25~25 μmol L^−1^ was used as the concentration gradient in subsequent experiments.

#### 2.5.2. Oxidative Damage Model of Endothelial Cells

To select a suitable H_2_O_2_ concentration, EA.hy926 cells were stimulated for 24 h at different H_2_O_2_ concentrations. The survival rate of the EA.hy926 endothelial cells decreased significantly with increasing H_2_O_2_ concentrations, as presented in Figure 9b. Based on the half lethal dose (IC_50_) principle, the model concentration of H_2_O_2_ in this experiment was selected as 700 μmol L^−1^.

#### 2.5.3. The Effects of Antioxidants on the Survival Rate of Oxidatively Damaged Endothelial Cells

Compared with the control group, the cell survival rate in the H_2_O_2_ group was significantly reduced (*p* < 0.01). Sea buckthorn compounds at concentrations of 6.25–25 μmol L^−1^ significantly increased the survival rate of EA.hy926 cells after H_2_O_2_ injury (*p* < 0.01) (Figure 9c).

#### 2.5.4. Hoechst 33,258 Staining Experiment

**Figure 10:**
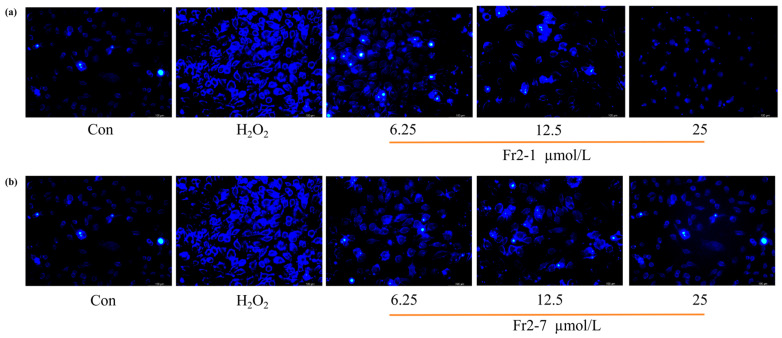
Effects of Fr2-1 and Fr2-7 on EA.hy926 cell apoptosis induced by H_2_O_2_ as detected by confocal microscopy (×100) (**a**,**b**). Note: densitometric data are presented as the means  ±  SEMs, *n*  =  3.

In the case of apoptosis, the nucleus was observed as dense or broken and dense. Hoechst 33,258 is a blue fluorescent dye with low toxicity. It can be absorbed and penetrated by cells without causing cell apoptosis. In this experiment, the protective effect of two sea buckthorn monomer compounds on EA.hy926 cells after 700 μmol L^−1^ H_2_O_2_ damage was assessed. As can be observed in Figure 10a,b, after staining by Hoechst 33,258, the blank-group samples all exhibited weak fluorescence, and the nuclei of the cells in the H_2_O_2_ group were densely and intensively stained with high-intensity fluorescence. Cells treated with 6.25 μmol L^−1^, 12.50 μmol L^−1^, and 25.00 μmol L^−1^ sea buckthorn compounds (Fr2-1 and Fr2-7) exhibited a dose-dependent reduction in nuclear fluorescence intensity and less condensed or fragmented nuclei, suggesting a protective effect against H_2_O_2_-induced nuclear damage.

### 2.6. Biochemical Analysis

**Figure 11:**
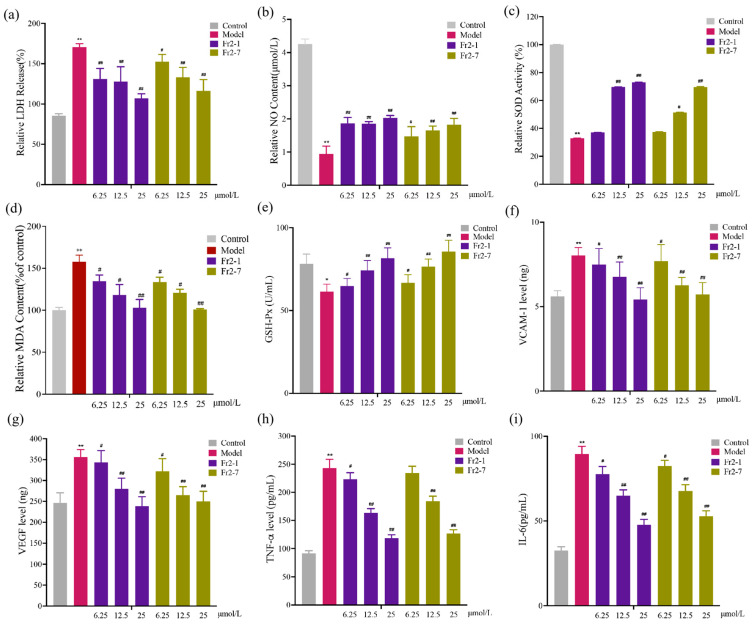
Effects of Fr2-1 and Fr2-7 on H_2_O_2_-injured EA.hy926 endothelial cells’ LDH release (**a**), NO content (**b**), SOD activity expression (**c**), relative MDA content (**d**), GSH-Px content (**e**), VCAM-1 level (**f**), VEGF level (**g**), TNF-α level (**h**), and IL-6 level (**i**). Note: densitometric data are presented as the means  ±  SEMs, *n*  =  3. Compared with the control group, * *p* < 0.05, and ** *p* < 0.01; compared with the model group, ^#^ *p* < 0.05, and ^##^ *p* < 0.01.

Under normal conditions, LDH cannot cross the membrane. However, when cells are damaged by oxidative stress, it alters the permeability of the cell membrane and releases LDH into the culture medium. Therefore, LDH release can be detected in the cell supernatant and, concurrently, provides a response to the extent of cellular damage. The LDH of the H_2_O_2_ group was higher than that of the control group (*p* < 0.01). This phenomenon indicated that the EA.hy926 endothelial cells were damaged. After treatment with the sea buckthorn compounds, the low concentration of Fr2-1 and Fr2-7 significantly reduced the LDH level (*p* < 0.01), and this reduction was more pronounced as the concentration increased (Figure 11a).

Previous studies have indicated that ROS decrease the generation and bioavailability of NO [27]. NO, an active nitrogen species, possesses potent vasodilatory and anti-inflammatory properties and inhibits platelet aggregation. It is an essential molecule that preserves vascular endothelial homeostasis. Reduced NO bioavailability constitutes a key component of endothelial dysfunction observed in cardiovascular pathologies. Endothelial cells regulate vascular tone, blood pressure, and vascular remodeling through the involvement of NO. NO production is impaired when the cells are exposed to OS. Compared with the control group, NO levels were significantly lower (*p* < 0.01) in the H_2_O_2_ group. At the same time, it was significantly higher (*p* < 0.01) in the EA.hy926 endothelial cells after treatment with the sea buckthorn compounds Fr2-1 and Fr2-7, as presented in Figure 11b. These results indicate that treatment with Fr2-1 and Fr2-7 was associated with increased NO levels compared to the H_2_O_2_-only group, suggesting a potential modulatory effect on NO bioavailability under oxidative stress conditions.

SOD is the main antioxidant enzyme involved in the endogenous scavenging of toxic free radicals that can scavenge ROS. Compared to the blank group, the activity of SOD in H_2_O_2_ group was significantly reduced (*p* < 0.05), and concentrations of Fr2-1 and Fr2-7 that were greater than 6.25 μmol L^−1^ increased the activity of SOD, as presented in Figure 11c. These results indicate that the sea buckthorn compounds Fr2-1 and Fr2-7 ameliorated the oxidative damage caused by H_2_O_2_ in the EA.hy926 endothelial cells. Similarly, H_2_O_2_-induced MDA levels were elevated, with MDA levels in the Fr2-1 and Fr2-7 groups being lower than in the H_2_O_2_ group (*p* < 0.05). MDA levels in Fr2-1 and Fr2-7 (25 μmol L^−1^) were slightly elevated in comparison to those in control group (*p* < 0.01) (Figure 11d). Intracellular GSH-Px levels were significantly reduced in the H_2_O_2_ group, whereas GSH-Px content was markedly elevated in the Fr2-1 and Fr2-7 groups at concentrations of 12.5 μmol L^−1^ and 25 μmol L^−1^, respectively (*p* < 0.01) (Figure 11e). SOD plays an important role in protecting cells from oxidative damage induced by ROS as well as inhibiting the oxidative inactivation of NO. It is well known that vascular endothelial homeostasis plays an important role in vascular functions. In specific situations, the vascular endothelium can lose its phenotype, leading to some cardiovascular pathological conditions [27]. The mechanism for the origin of endothelial dysfunction is proposed to be the enhanced production of reactive oxygen species. The MDA level is closely associated with several components of metabolic syndrome [28]. This study assessed the activity of antioxidant enzymes (SOD and GSH-Px) and the level of the oxidative metabolite MDA. Compared to the control group, Fr2-1 and Fr2-7 increased SOD activity while significantly reducing MDA levels. However, Fr2-1 and Fr2-7 significantly increased the activity of the antioxidant enzyme GSH-PX in the cell. A previous study indicated that flavonoids have the ability to effectively reduce oxidative stress [29]. Therefore, Fr2-1 and Fr2-7 treatment led to increased SOD and GSH-Px activities and decreased MDA content, indicating that these compounds may mitigate oxidative stress-related changes in these enzymatic markers.

The H_2_O_2_ group had significantly higher levels of VCAM-1 and VEGF in comparison to those in the control group (*p* < 0.01). However, Fr2-1 and Fr2-7 decreased the levels of VCAM-1 and VEGF in a dose-dependent manner. The level of VCAM-1 and VEGF in the the Fr2-1 and Fr2-7 groups of 12.5 μmol L^−1^ and 25 μmol L^−1^ was significantly lower than that in the H_2_O_2_ group (*p* < 0.01) (Figure 11f,g). VEGF directly acts on vascular skin cells, can promote the division and proliferation of vascular endothelial cells, can promote the formation of new blood vessels, and can strongly increase the permeability of blood vessels [30]. VCAM-1 expressed on vascular endothelial cells can promote the adhesion of white blood cells and vascular endothelial cells, and accelerate the downstream movement of white blood cells to vascular endothelial cells [31,32]. The results indicate that Fr2-1 and Fr2-7 have not yet been shown to directly regulate endothelial function via VEGF and VCAM-1 but suggest a potential association between the two.

TNF-α and IL-6 levels in the H_2_O_2_ group were significantly higher than in the control group (*p* < 0.01). However, Fr2-1 and Fr2-7 decreased the levels of TNF-α and IL-6 in a dose-dependent manner. The level of TNF-α and IL-6 in the the Fr2-1 and Fr2-7 groups of 12.5 μmol L^−1^ and 25 μmol L^−1^ was significantly lower than that in the H_2_O_2_ group (*p* < 0.01) (Figure 11h,i). In addition to the ROS and oxidative stress mentioned earlier, the inflammatory response is considered to be a common pathogenic factor in vascular impairment [33]. In order to investigate the possible involvement of the molecular mechanism of Fr2-1 and Fr2-7 in endothelial dysfunction, the current study evaluated the expression of the inflammatory cytokines IL-6 and TNF-α. The levels of TNF-α and IL-6 in the H_2_O_2_ group were significantly higher than those in the control group. However, Fr2-1 and Fr2-7 reduced the levels of TNF-α and IL-6 in a dose-independent manner. This suggests that vascular protection by Fr2-1 and Fr2-7 may be related to anti-inflammatory activity since it reduces the levels of TNF-α and IL-6.

Here, the findings indicate a molecular mechanism underlying the therapeutic effects of Fr2-1 and Fr2-7, and they highlight a new therapeutic intervention for preventing endothelial dysfunction induced by H_2_O_2_.

## 3. Discussion

In this study, an online HPLC-DPPH assay system combined with medium- and high-pressure chromatographic separation was used to screen and isolate antioxidant constituents from sea buckthorn. Using this activity-guided strategy, three compounds, namely tetrahydroharmol (Fr2-1), manghaslin (Fr2-5), and isorhamnetin 3-O-(6-O-E-sinapoyl)-β-D-glucopyranosyl-(1-2)-β-D-glucopyranoside-7-O-α-L-rhamnopyranoside (Fr2-7), were isolated from Fr2, and Fr2-7 was isolated from sea buckthorn for the first time. Fr2-1 is an alkaloid, while Fr2-5 and Fr2-7 are flavonoids. The purity of all monomeric compounds isolated from Fr2 exceeded 95%. Their antioxidant activity was evaluated using DPPH radical scavenging assays. The results indicate that the compounds Fr2-1, Fr2-5, and Fr2-7 exhibit good antioxidant activity, demonstrating the effectiveness of this separation method for isolating antioxidant compounds. However, due to the limited amount of purified Fr2-5 obtained after preparative isolation, this compound was not included in the subsequent cell-based assays, and the biological evaluation was therefore focused on Fr2-1 and Fr2-7. These results demonstrate that an online HPLC-DPPH activity screening system is of practical value for the rapid identification and purification of antioxidant components from complex plant extracts.

The present findings also indicate that an online HPLC-DPPH system is an efficient activity-guided screening approach for identifying antioxidant candidates from sea buckthorn. At the same time, the response observed in this system may be influenced by chromatographic behavior, compound concentration, online mixing efficiency, and the reaction kinetics between individual compounds and DPPH radicals. Therefore, this strategy is especially valuable for rapid screening and targeted isolation, while complementary off-line assays remain helpful for broader antioxidant evaluation [34,35].

Using molecular docking simulations, the binding affinities of Fr2-1 and Fr2-7 for five target proteins (LDH, SOD, Nrf2, iNOS, and eNOS) were predicted. The calculated binding energies were all below −5 kcal mol^−1^, a threshold typically used to indicate potential spontaneous binding. These results suggest that Fr2-1 and Fr2-7 theoretically possess the ability to interact with these proteins. However, molecular docking is a predictive computational simulation method and cannot demonstrate actual binding or functional regulation in living cells. The predicted interactions merely provide a direction for subsequent mechanistic studies and do not constitute direct evidence of target binding, enzyme inhibition, or pathway activation, and further validation through in vivo experiments is required [36,37].

In the hydrogen peroxide-induced oxidative damage model of EA.hy926 cells, Fr2-1 and Fr2-7 increased cell survival rates. Hoechst staining revealed that nuclear condensation and fragmentation were less frequent in the groups treated with these compounds, indicating that they exert a protective effect against hydrogen peroxide-induced nuclear damage. However, this method provides only a qualitative assessment of apoptosis and cannot accurately distinguish between early apoptosis, late apoptosis, and necrosis, and quantitative methods such as flow cytometry are required to confirm the compound’s anti-apoptotic effects [38,39]. Furthermore, compared with the H_2_O_2_ model group, both compounds reduced lactate dehydrogenase (LDH) release and modulated multiple markers associated with oxidative stress, including increased levels of nitric oxide (NO), superoxide dismutase (SOD), and glutathione peroxidase (GSH-Px), and reduced malondialdehyde (MDA) levels. Together, these results suggest that Fr2-1 and Fr2-7 may alleviate H_2_O_2_-induced oxidative injury and improve the cellular redox-related state in EA.hy926 cells.

Fr2-1 and Fr2-7 also decreased the levels of VCAM-1, VEGF, TNF-α, and IL-6 in the cell model. Since these markers are associated with endothelial activation, inflammation, and stress responses, their reduction suggests that the two compounds may modulate oxidative stress-related inflammatory signaling under the present experimental conditions. Thus, beyond their radical scavenging activity in the chemical assay, these two compounds also showed favorable effects on multiple injury-related biochemical indicators in the endothelial cell model.

The biochemical markers measured in this study provide a coherent picture of the cellular response to treatment. LDH release reflects membrane damage, NO content reflects nitric oxide-related metabolism or bioavailability, SOD and GSH-Px represent antioxidant enzyme-associated responses, and MDA is an indicator of lipid peroxidation. The coordinated changes observed across these indicators suggest that Fr2-1 and Fr2-7 were associated with attenuation of oxidative stress-related cellular disturbances induced by H_2_O_2_. However, because intracellular ROS levels, mitochondrial function, apoptosis pathways, and upstream signaling events were not directly measured, the underlying mechanism remains unresolved.

It is also important to place the present findings in the context of previous studies on sea buckthorn extracts. In our earlier work, sea buckthorn polyphenol extracts showed protective effects against vascular endothelial dysfunction in hyperlipidemic rats, including modulation of antioxidant enzyme activity, inflammatory cytokines, and vascular endothelial markers in aortic tissue. In contrast, the present study focused on the isolation of individual antioxidant constituents and their preliminary evaluation in an H_2_O_2_-injured EA.hy926 cell model. Therefore, the current work can be viewed as a follow-up study that helps identify candidate monomeric compounds potentially contributing to the previously observed extract-level bioactivity, and it provides a basis for linking the antioxidant activity of sea buckthorn extracts with specific active constituents [40].

Several limitations of this study should be acknowledged. First, the biological evaluation was performed only in a single H_2_O_2_-induced EA.hy926 cell model, which represents a simplified in vitro oxidative injury system. Second, most readouts were indirect biochemical or immunological markers, and no direct mechanistic validation was conducted. Third, Hoechst staining provided only qualitative morphological evidence and cannot distinguish apoptosis from other forms of cell death. Fourth, no in vivo experiments were performed; therefore, the relevance of these findings to vascular physiology or cardiovascular protection remains to be established.

Overall, the present study demonstrates that the online HPLC-DPPH-guided strategy is effective for isolating antioxidant compounds from sea buckthorn and provides preliminary evidence that Fr2-1 and Fr2-7 are associated with reduced oxidative stress-related injury in H_2_O_2_-treated EA.hy926 cells. Further studies incorporating direct ROS assays, quantitative apoptosis analysis, pathway validation, and in vivo models are needed to clarify the mechanisms and physiological relevance of these observations.

## 4. Materials and Methods

### 4.1. Chemicals and Reagents

An online HPLC-DPPH identification system was established using LC10AD and LC16 HPLC (Shimadzu, Kyoto, Japan). An electrospray mass spectrometer (Waters, Milford, MA, USA) was used for analysis, and ^1^H and ^13^C NMR spectra were obtained on a Bruker Avance 600 MHz instrument (Bruker, Karlsruhe, Germany) using tetram-ethylsilane as the internal standard and MeOH-d4 as the solvent. Confocal microscopy was performed using a Zeiss LSM 880 confocal microscope (Carl Zeiss, Oberkochen, Germany). The UV absorbance was determined using a Readmax 1900 enzyme-linked immunosorbent assay (Shanghai Shanpu Biotechnology Co., Ltd., Shanghai, China). Polyamide (100–200 mesh) for the medium-pressure column was purchased from Changfeng Chemical Co., Ltd. (Changzhou, Jiangsu, China). ReproSil-Pur C18 AQ columns (4.6 × 250 mm, 5 μm; 20 × 250 mm, 5 μm) were purchased from Maisch Corporation (Maisch, Baden, Wuerttemberg, Germany). DPPH was purchased from Sigma-Aldrich (Aldrich, Steinheim, Germany). Analytical-grade ethanol, methanol (MeOH), dichloromethane (CH_2_Cl_2_), acetonitrile (ACN), n-hexane, and ethyl acetate were purchased from the Kelong Chemical Reagent Factory (Chengdu, Sichuan, China). HPLC-grade ethanol, MeOH, and acetonitrile (ACN) were purchased from the Kelong Chemical Reagent Factory (Chengdu, Sichuan, China). HPLC-grade H_2_O was prepared using a water purifier (Moore, Chongqing, China). Dulbecco’s modified eagle medium (DMEM), fetal bovine serum (FBS), phosphate buffered saline (PBS), trypsin, and penicillin were obtained from Procell Life Science & Technology Co., Ltd. (Wuhan, Hubei, China). A BCA protein concentration assay kit and Hoechst 33,258 staining solution were purchased from Beyotime Biotechnology Co., Ltd. (Shanghai, China). A multi-functional enzyme marker (TECAN, Männedorf, Switzerland) was used for the detection of each kit.

### 4.2. Plant Material

In September 2019, sea buckthorn fruits from Zhamao village (35°20′55″ N, 101°54′14″ E, altitude 2910 m), Tongren County, Qinghai Province, were collected and identified as Chinese sea buckthorn (*Hippophae rhamnoides* Linn. subsp. Sinensis Rousi) by Associate Professor Yang Shibing of Qinghai University. The specimen was deposited under the herbarium of the pharmacy department of Qinghai University in Xining, China (Under the voucher number 6323210517002LY.)

### 4.3. Extraction and Pretreatment of Samples

The collected sea buckthorn fruits were juiced, filtered and dried to obtain pomace (4.0 kg), which was ultrasonically extracted with 70% ethanol at 60 °C for 30 min (at a solid–liquid ratio of 1:10, at 60 °C, and at an ultrasonic power of 1000 W). After centrifugation, the supernatant was collected and concentrated using a rotary evaporator at 50 °C. The concentrate was decontaminated using a column packed with AB-8 macroporous resin. The ethanol eluent in the column was desorbed with 95% ethanol, concentrated at 50 °C using a vacuum rotary evaporator, and dried at 60 °C in an oven. Finally, 120.0 g of dried samples were obtained. Sea buckthorn extract (60.0 g) was dissolved in methanol, mixed with polyamide (120.0 g) gel and dried in an oven at 55 °C.

The mixture (25.0 g) was loaded into a small medium-pressure chromatographic column (49 × 100 mm), which was connected with a polyamide medium-pressure column (49 × 460 mm) and a preparative liquid chromatogram for sample pretreatment. The sample was eluted with a water/methanol system. The linear elution program was 0–270 min (0–100% methanol), the flow rate was 57.0 mL min^−1^, the chromatogram was recorded at 210 nm wavelengths, the sample volume was 25g each time, and the sample was repeated 6 times. Finally, five fractions (marked as Fr1, Fr2, Fr3, Fr4 and Fr5) were obtained.

### 4.4. DPPH Assay to Evaluate the Active Ingredients

The DPPH scavenging experiment was carried out in 96-well plates. The sample solution of each component and the blank solution (50% ethanol aqueous solution) were diluted, and 30.0 μL was added to the 96-well plate, and then 70.0 μL of the prepared DPPH solution was added. The 50% ethanol aqueous solution was used as the blank control solution, and three repeated holes were set for each concentration. The 96-well plates were placed in an oscillating incubator and incubated in the dark for 30 min. The absorbance (OD value) was detected at a wavelength of 517 nm using a microplate reader. Each sample was measured three times in parallel. The DPPH clearance rate calculation formula is as follows:

$$\mathrm{DPPH}\mathrm{clearance}=[1-(\frac{A_{1}-A_{2}}{A_{3}-A_{2}})]\times 100\%$$

Here, A_1_ is the absorbance of the sample solution, A_2_ is the absorbance of 50% ethanol–water as a blank solution, and A_3_ represents the absorbance of 50% ethanol–water instead of the sample as a control solution.

### 4.5. Fr2 Antioxidants Targeted Separation

Two high-performance liquid chromatographs were connected (Shimadzu LC-16 and Shimadzu LC10AD). The Shimadzu LC-16 system was used for conventional high-performance liquid chromatography analysis, and the DPPH solution was pumped by the Shimadzu LC10AD pump at a constant flow rate. Two high-performance liquid chromatographs were connected by a three-way valve and an 18 m long PEEK pipeline with a radius of 0.25 μm. The sample solution was detected by the Shimadzu LC-16 system at a wavelength of 210 nm and then flowed into the Shimadzu LC10AD pump and DPPH solution to be fully mixed in the PEEK pipeline for detection at a wavelength of 517 nm.

The potential antioxidant activity peaks in Fr2 were identified and prepared using an online HPLC-DPPH activity screening system. The LC16 high-performance liquid chromatography analysis conditions were as follows: ReproSil-Pur C18 AQ (4.6 × 250mm, 5 μm) analytical column, mobile phase A of 0.1% formic acid water, mobile phase B of chromatographic acetonitrile, gradient elution, 0–60 min, 5–25% B, flow rate of 1 mL min^−1^, and chromatography at a wavelength of 210 nm. Preparation conditions: the ReproSil-Pur C18 AQ reversed-phase column (20 × 250 mm, 5 μm) was used, and the other conditions were the same as above. The mobile phase of the LC10AD high-performance liquid chromatography was the DPPH ethanol solution, the flow rate was set at 0.8 mL min^−1^, and the detection wavelength was set at 517 nm.

The seven collected components were reanalyzed according to the analytical chromatographic conditions. Finally, the antioxidant activities of Fr2-1 and Fr2-7 were verified using an online HPLC-DPPH activity screening system.

### 4.6. Molecular Docking

The Chem 3D software (Chem 3D 22.0.0.22) was used to generate and optimize the minimum binding energy, and the mol2 file of the ligand small molecule was derived. Three-dimensional structures of five target proteins (receptors) were retrieved and downloaded from the RCSB protein database (https://www.rcsb.org/ accessed on 25 February 2025). The PyMOL software (PyMOL Molecular Graphics System 2.2.0) was used to remove the water molecules and small molecules of the receptor, and they were saved as pdb files. Autodock (AutoDockTools 1.5.7) was used to hydrogenate the target protein to determine the coordinates of the active pocket box of the target protein. After correctly generating gpf and dpf files, the Autodock software was run, and a visual analysis of the optimal conformation was performed through PyMOL.

### 4.7. Fr2 Antioxidant Monomer In Vitro Antioxidant Evaluation

#### 4.7.1. Cell Culture

EA.hy926 human umbilical vein endothelial fusion cells were cultured in DMEM medium containing 20% fetal bovine serum, 100 U mL^−1^ of penicillin and 100 mg mL^−1^ of streptomycin. The cells were placed in a constant-temperature incubator containing atmospheric air and 5% CO_2_ at 37 °C for subculture.

#### 4.7.2. Assessment of Endothelial Cell Viability

The concentration of the EA.hy926 endothelial cells in the logarithmic growth phase was adjusted to 5 × 10^4^ cells per well, and then 100 μL of each well was inoculated into 96-well plates and cultured for 24 h. The original medium was replaced with a sea buckthorn compound at a concentration of 0-100 μmol L^−1^ and then cultured for 24 h. The absorbance (A) was measured at 490 nm using the MTT method. The cell survival rate was calculated. Cell survival rate = treatment group A value/blank group A value × 100%.

#### 4.7.3. Establishment of Endothelial Cell Oxidative Damage Model

Cells in the log-growth stage were selected for further analysis. EA.hy926 cells were inoculated into 96-well plates, and the cells completely adhered to the cell walls 24 h after inoculation. Eleven H_2_O_2_ concentrations of 0~1000 μmol L^−1^ were added into 96-well plates in a serum-free medium. After inoculating for 24 h, MTT was used to measure absorbance at 490 nm to calculate the survival ratio of cells and to determine the optimum H_2_O_2_ concentration for modeling.

#### 4.7.4. Effects of Antioxidants on the Survival of Oxidatively Damaged Endothelial Cells

The effects of five components on the activity of EA.hy926 cells were detected by MTT assay. In this experiment, seven concentration gradients were set for each component, i.e., 0, 10, 20, 50, 100, 150, and 200 μg mL^−1^, respectively, and 6 replicates were set for each concentration. After the cells were treated with a drug concentration for 24 h, the absorption value was read at 490 nm using a microplate reader.

EA.hy926 cells were seeded into 96-well plates, and the cells were completely adherent 24 h after inoculation. The experiment was divided into the control group, H_2_O_2_ group and H_2_O_2_ + different concentrations of sea buckthorn compound group. After 24 h of treatment, MTT assay was used to detect the absorbance at 490 nm, and the cell viability was calculated.

#### 4.7.5. Hoechst 33,258 Staining

The experiment was divided into blank, H_2_O_2_, and H_2_O_2_ + different concentrations of sea buckthorn compounds groups. The cells were incubated for 24 h to induce apoptosis, and the cell culture solution in the 6-well plates of each group was discarded. A total of 1.0 mL of the Hoechst 33,258 staining solution was added to each well, the cells to be stained were completely covered and placed in a 5% CO_2_ cell culture incubator for 30 min, the staining solution was discarded, the cells were washed three times with PBS for one minute, and the PBS was discarded. The cell morphology was observed with an inverted bio microscope.

#### 4.7.6. Biochemical Analysis of EA.hy926 Endothelial Cells

EA.hy926 cells were inoculated into 6-well plates; fully adhered to the wall 24 h after inoculation; and divided into blank, H_2_O_2_, and H_2_O_2_ + different concentrations of sea buckthorn compounds groups. After 24 h of culture, the cells and supernatant were collected for various biochemical assays. LDH, NO, SOD, MDA and GSH-Px kits were purchased from Jiancheng Biotechnology (Nanjing, China). Protein concentrations in cell lysates were determined using the BCA method for standardization, and the following parameters were measured:

Cell collection method: EA.hy926 cells are adherent cells. Remove the supernatant, digest with 0.25% trypsin at room temperature for 2 min, add a culture medium to stop the digestion, gently pipette up and down with a micropipette, aspirate all the liquid into an EP tube, centrifuge at 1000 rpm for 10 min, discard the supernatant, add 0.3 mL of 0.1 mol L^−1^, add a pH 7–7.4 phosphate buffer to resuspend the cells, sonicate in an ice-water bath (power 200–300 W, 5 s on, 15 s off, repeated 3 times), and set aside.

BCA protein assay: A protein standard (0.5 mg mL^−1^ bovine serum albumin) was added to a 96-well plate at volumes of 0, 1, 2, 4, 8, 12, 16, and 20 µL, each adjusted to 20 µL with a diluent. Sample wells received an appropriate volume of lysate (adjusted to 20 µL with a diluent). A BCA working solution (200 µL, prepared at a 50:1 ratio of reagent A to B) was added to each well and incubated at 37 °C for 20–30 min. Absorbance was measured at 562 nm, and protein concentrations were calculated from a standard curve.

Lactate dehydrogenase (LDH) leakage: Follow the instructions in the kit manual to perform the assay. First, prepare the necessary working solutions, dissolve one vial of Coenzyme I powder in 1.3 mL of double-distilled water to create the Coenzyme I solution, dilute the 4 mol L^−1^ NaOH solution 10-fold with double-distilled water to prepare a 0.4 mol L^−1^ NaOH solution. Dilute the 2 μmol mL^−1^ sodium pyruvate standard solution 10-fold with double-distilled water to prepare a 0.2 μmol mL^−1^ sodium pyruvate standard solution. Set up blank wells, standard wells, test wells, and control wells in a 96-well plate. Add 20 μL of double-distilled water to the blank wells, add 4 μL of double-distilled water and 16 μL of the 0.2 μmol mL^−1^ sodium pyruvate standard solution to the standard wells, add 16 μL of the sample to be tested to the test wells, and add 4 μL of double-distilled water and 16 μL of the sample to be tested to the control wells. Next, add 20 μL of the matrix buffer to each well. Add an additional 4 μL of the coenzyme I solution to the sample wells, but do not add any to the control wells. Gently shake to mix, then incubate at 37 °C for 15 min. Then, add 20 μL of 2,4-dinitrophenylhydrazine to each well, shake again to mix, and incubate at 37 °C for 15 min. Finally, add 200 μL of the 0.4 mol L^−1^ NaOH solution to each well, shake to mix, and let it stand at room temperature for 5 min. Measure the absorbance of each well at a wavelength of 440 nm using a microplate reader. LDH activity (U g^−1^ protein) was calculated as follows:

$$LDH=\frac{A_{4}-A_{3}}{A_{2}-A_{1}}\times \frac{C}{Cpr}$$

Here, A_4_ is the absorbance value of the sample well, A_3_ is the absorbance value of the control well, A_2_ is the absorbance value of the standard well, A_1_ is the absorbance value of the blank well, C is the concentration of the standard solution (0.2 μmol mL^−1^), and Cpr is the protein concentration of the sample (gprot mL^−1^; “prot” refers to protein).

Nitric oxide (NO) content: Perform the assay according to the kit instructions. Prepare the color developer fresh by mixing Reagent 3, Reagent 4, and Reagent 5 in a volume ratio of 2:5:1:1. Dilute the 2 mmol L^−1^ sodium nitrite standard solution 100-fold with distilled water to prepare a 20 μmol L^−1^ sodium nitrite standard solution, and prepare this solution fresh. Set up blank wells, standard wells, and test wells in a 96-well plate, and add 0.16 mL of double-distilled water to the blank wells, 0.16 mL of the 20 μmol L^−1^ sodium nitrite standard solution to the standard wells, and 0.16 mL of the supernatant to be tested to the test wells. Add 0.08 mL of color developer to each well, mix the well, and let it stand at room temperature for 15 min. Measure the absorbance of each well at a wavelength of 550 nm using a microplate reader. NO content (µmol g^−1^ protein) was calculated as follows:

$$NO=\frac{A_{3}-A_{1}}{A_{2}-A_{1}}\times C\times N÷Cpr$$

Here, A_3_ is the absorbance value of the sample well, A_2_ is the absorbance value of the standard well, A_1_ is the absorbance value of the blank well, C is the concentration of the standard solution (20 μmol mL^−1^), N is the dilution factor, and Cpr is the sample protein concentration (gprot mL^−1^; “prot” refers to protein).

Superoxide dismutase (SOD) activity: Perform the assay according to the kit instructions. Prepare the required working solutions prior to the experiment, dilute Reagent 1 (substrate stock solution) with double-distilled water at a 1:9 ratio to obtain the substrate working solution, dilute Reagent 4 (stock solution) at a 1:14 ratio to obtain the catalyst working solution, and mix Reagents 5 and 6 with glacial acetic acid in a 3:3:2 volume ratio to prepare the color development solution. Set up the control and test tubes separately for total SOD (T-SOD) and copper–zinc SOD (CuZn-SOD). Add 1.0 mL of the substrate working solution to each tube. Then, add double-distilled water to the T-SOD control tube, the sample to be tested to the T-SOD test tube, the control supernatant to the CuZn-SOD control tube, and the sample supernatant to the CuZn-SOD test tube. Subsequently, add 0.1 mL of Reagent 2 (enzyme working solution), 0.1 mL of Reagent 3 (hydroxylamine solution), and 0.1 mL of the catalyst working solution to each tube in sequence. Vortex to mix thoroughly, then incubate at 37 °C for 40 min. Finally, add 2 mL of the color development solution, mix well, and let the sample stand at room temperature for 10 min. Measure the absorbance at a wavelength of 550 nm. SOD activity (U mL^−1^) was calculated as follows:

$$SOD=\frac{A_{1}-A_{2}}{A_{1}}÷50\%\times \frac{V_{1}}{V_{2}}\times N$$

Here, A_2_ is the absorbance value of the sample well, A_1_ is the absorbance value of the control well, V_1_ is the total volume of the reaction mixture, V_2_ is the sample volume, and N is the dilution factor.

Malondialdehyde (MDA): Malondialdehyde (MDA), a degradation product of lipid peroxides, can condense with thiobarbituric acid (TBA) to form a red product with a maximum absorption peak at 532 nm. Since thiobarbituric acid (TBA) serves as the substrate, this method is referred to as the TBA method. Perform the assay according to the kit instructions. Prior to the experiment, prepare the required working solutions. Dissolve Reagent 2 (substrate powder) in distilled water according to the specified ratio to prepare the substrate working solution. Dissolve Reagent 3 (color developer powder) in distilled water by heating to 90–100 °C, then add glacial acetic acid to prepare the color developer working solution. Prepare blank tubes, standard tubes, test tubes, and control tubes. Add 0.2 mL each of the 10 nmol mL^−1^ tetraethoxypropane standard and anhydrous ethanol to the blank tubes; add only 0.2 mL of the standard to the standard tubes, and add 0.2 mL of the sample to be tested to both the test tubes and control tubes. Add 0.2 mL of Reagent 1 (reaction buffer) to all tubes, and mix thoroughly. Next, add 3 mL of substrate solution to each tube. Add 1 mL of the color development solution to the blank, standard, and test tubes, and add 1 mL of 50% glacial acetic acid to the control tube. After mixing, place the tubes in a boiling water bath at 95 °C or higher for 40 min. After cooling, centrifuge at 4000 rpm for 10 min, and measure the absorbance of the supernatant at a wavelength of 532 nm. The MDA content (nmol mg^−1^ protein) was calculated using the following formula:

$$MDA=\frac{A_{4}-A_{3}}{A_{2}-A_{1}}\times \frac{C}{Cpr}$$

Here, A_4_ is the absorbance value of the sample well, A_3_ is the absorbance value of the control well, A_2_ is the absorbance value of the standard well, A_1_ is the absorbance value of the blank well, C is the concentration of the standard solution (0.2 μmol mL^−1^), and Cpr is the sample protein concentration (gprot mL^−1^; “prot” refers to protein).

Glutathione peroxidase (GSH-Px): Perform the assay according to the kit instructions. Prepare the necessary working solutions before the experiment, dilute Reagent 1 (substrate stock solution) 100-fold with distilled water to serve as the substrate working solution. Prepare the Reagent 2 working solution by mixing Powder A and Solution B according to the instructions. Dissolve the Reagent 3 powder in distilled water, and dissolve the Reagent 5 powder in distilled water to serve as the Reagent 5 working solution. Prepare a 1 mmol L^−1^ of the GSH standard solution using the GSH standard dilution buffer, then dilute it further to a 20 μmol L^−1^ GSH standard solution. Enzymatic reaction: Take a non-enzyme tube and an enzyme tube, and add 0.2 mL of 1 mmol L^−1^ GSH to each. Add 0.2 mL of the sample homogenate to the enzyme tube, and do not add anything to the non-enzyme tube. After pre-warming at 37 °C for 5 min, add 0.1 mL of the substrate working solution to each tube, and allow it to react precisely at 37 °C for 5 min. Immediately add 2 mL of the Reagent 2 working solution, add 0.2 mL of the sample homogenate to the non-enzyme tube, leave the enzyme tube empty, mix well, then centrifuge at 3500–4000 rpm for 10 min, and collect the supernatant. Colorimetric reaction: Take a blank tube (add 1 mL of the standard dilution solution), the standard tube (containing 1 mL of the 20 μmol L^−1^ GSH standard solution), the non-enzyme tube, and the enzyme tube (each containing 1 mL of supernatant). Add 1 mL of the Reagent 3 working solution, 0.25 mL of the Reagent 4 working solution, and 0.05 mL of the Reagent 5 working solution to each tube. Mix well, let the sample stand at room temperature for 15 min, and measure the absorbance at a wavelength of 412 nm. The GHS-Px activity (U mg^−1^ protein) was calculated using the following formula:

$$GSH-Px=\frac{A_{4}-A_{3}}{A_{2}-A_{1}}\times C\times N÷T÷(V\times Cpr)$$

Here, A_4_ is the absorbance value of the non-enzyme tube, A_3_ is the absorbance value of the enzyme tube, A_2_ is the absorbance value of the standard tube, A_1_ is the absorbance value of the blank tube, C is the concentration of the GSH standard solution in the colorimetric reaction, N is the dilution factor of the enzymatic reaction system, T is the enzymatic reaction time, V is the sample volume for the enzymatic reaction, and Cpr is the protein concentration of the homogenate (mgprot mL^−1^; “prot” refers to protein).

Cytokine and adhesion molecule measurements (IL-6, TNF-α, VCAM-1, and VEGF): The levels of interleukin-6 (IL-6), tumor necrosis factor alpha (TNF-α), vascular cell adhesion molecule-1 (VCAM-1), and vascular endothelial growth factor (VEGF) were determined using commercial sandwich ELISA kits (Beyotime Biotech, Shanghai, China) [41,42]. Cell culture supernatants were collected by low-speed centrifugation. Briefly, 100 µL of the standard or sample was added to pre-coated wells and incubated at room temperature for 120 min. After five washes, 100 µL of a biotinylated detection antibody was added and incubated for 60 min. Following another wash step, 100 µL of HRP–streptavidin was added and incubated for 20 min in the dark. After washing, 100 µL of the TMB substrate was added and incubated for 20 min in the dark. The reaction was stopped with 50 µL of a stop solution, and absorbance was measured at 450 nm using a microplate reader. Cytokine concentrations were calculated from standard curves. Protein concentrations were determined by BCA assay for normalization.

### 4.8. Data Analysis

Each experimental replication was performed independently three times. GraphPad Prism 8.0 was used for statistical analysis, and the data are expressed as means ± standard errors of the mean (SEM). A one-way ANOVA was performed among the groups with * *p <* 0.05 considered as statistically significant.

## 5. Conclusions

In conclusion, this study established an antioxidant-activity-guided isolation method for sea buckthorn fruit, utilizing a combined medium- and high-pressure chromatography system with HPLC-DPPH screening. Three antioxidant compounds were preliminarily isolated: Tetrahydroharmol (Fr2-1), Manghaslin (Fr2-5) and Isorhamnetin 3-O-(6-O-E-sinapoyl)-β-D-glucopyranosyl-(1-2)-β-D-glucopyranoside 7-O-α-L-rhamnopyranoside (Fr2-7), with Fr2-7 being isolated from sea buckthorn for the first time. Fr2-1 and Fr2-7 can modulate various markers associated with oxidative stress and inflammation in an oxidatively damaged endothelial cell model, suggesting that sea buckthorn-derived compounds have the potential to serve as natural antioxidant candidates, while also validating the effectiveness of an online HPLC-DPPH activity screening system. However, because the present study relied on a single in vitro model and indirect biochemical markers without direct pathway validation or in vivo evidence, the physiological significance and mechanisms of these effects require further investigation.

## Figures and Tables

**Table 1:** The identification results of sea buckthorn fruit extract.

Label	Compound	Formula	Mass (Da)	*m*/*z* (Precursor Ion)	Adducts	Reference
1	Tetrahydroharmol	C_12_H_14_N_2_O	202.11	201.11	-H	[19]
2	Manghaslin	C_33_H_40_O_20_	756.55	755.55	-H	[20]
3	Isorhamnetin 3-O-(6-O-E-sinapoyl)-D-glucopyranosyl-(1-2)-β-D- glucopyranoside7-O-α-L- rhamnopyranoside	C_45_H_52_O_25_	992.39	991.39	-H	[21]

Note: Reference, i.e., relevant research literature and standard, data were referenced on behalf of the constituents, so as to screen and analyze the constituents of sea buckthorn fruit extract.

**Table 2:** Molecular docking parameters and results.

Protein	Ligand	Number of Points	Center Grid Box	Docking Score
LDH ID:7DBJ	Fr2-1 Fr2-7	X_dimension = 100 Y_dimension = 100 Z_dimension = 100	X_center = 14.254 Y_center = 6.991 Z_center = 8.793	−7.36 kcal mol^−1^ −5.7 kcal mol^−1^
SOD ID:4MCM	Fr2-1 Fr2-7	X_dimension = 100 Y_dimension = 100 Z_dimension = 100	X_center = 39.142 Y_center = 84.311 Z_center= 46.69	−8.2 kcal mol^−1^ −4.37 kcal mol^−1^
Nrf2 ID:2FLU	Fr2-1 Fr2-7	X_dimension = 105 Y_dimension = 110 Z_dimension = 90	X_center = 16.626 Y_center = 17.737 Z_center= 6.762	−7.16 kcal mol^−1^ −8.84 kcal mol^−1^
iNOS ID:3E7G	Fr2-1 Fr2-7	X_dimension = 126 Y_dimension = 84 Z_dimension = 126	X_center = 46.206 Y_center = 22.9 Z_center= 54.584	−5.94 kcal mol^−1^ −6.69 kcal mol^−1^
eNOS ID:3NOS	Fr2-1 Fr2-7	X_dimension = 95 Y_dimension = 100 Z_dimension = 110	X_center = 16.166 Y_center = 15.32 Z_center= 40.154	−7.17 kcal mol^−1^ −7.29 kcal mol^−1^

## Data Availability

The datasets supporting the conclusions of this article are included within the manuscript.

## Supplementary Materials

The following supporting information can be downloaded at: https://www.mdpi.com/article/10.3390/ijms27093757/s1.

## Abbreviations

The following abbreviations are used in this manuscript:

CVD	Cardiovascular disease
OS	Oxidative stress
DPPH	1,1-diphenyl-2-picrylhydrazyl
EtOH	ethanol
MeOH	methanol
ACN	acetonitrile
MPLC	medium-pressure liquid chromatography
ANOVA	analysis of variance
^1^H NMR	^1^H-nuclear magnetic resonance
^13^C NMR	^13^C-nuclear magnetic resonance
NO	nitric oxide
SOD	superoxide dismutase
LDH	lactate dehydrogenase
PBS	phosphate buffered saline
FBS	fetal bovine serum
IL-6	interleukin-6
TNF-α	tumor necrosis factor alpha
GSH-Px	glutathione peroxidase
VCAM-1	vascular cell adhesion molecule-1
VEGF	vascular endothelial growth factor
