# Supplementary figures and images for: Preparative Separation of Antioxidants from Sea Buckthorn and Its Antioxidant Activity In Vitro via Endothelial Function Regulation
Source: Int J Mol Sci. 2026 Apr 23;27(9):3757. doi: 10.3390/ijms27093757 (PMC13163278; doi:10.3390/ijms27093757)

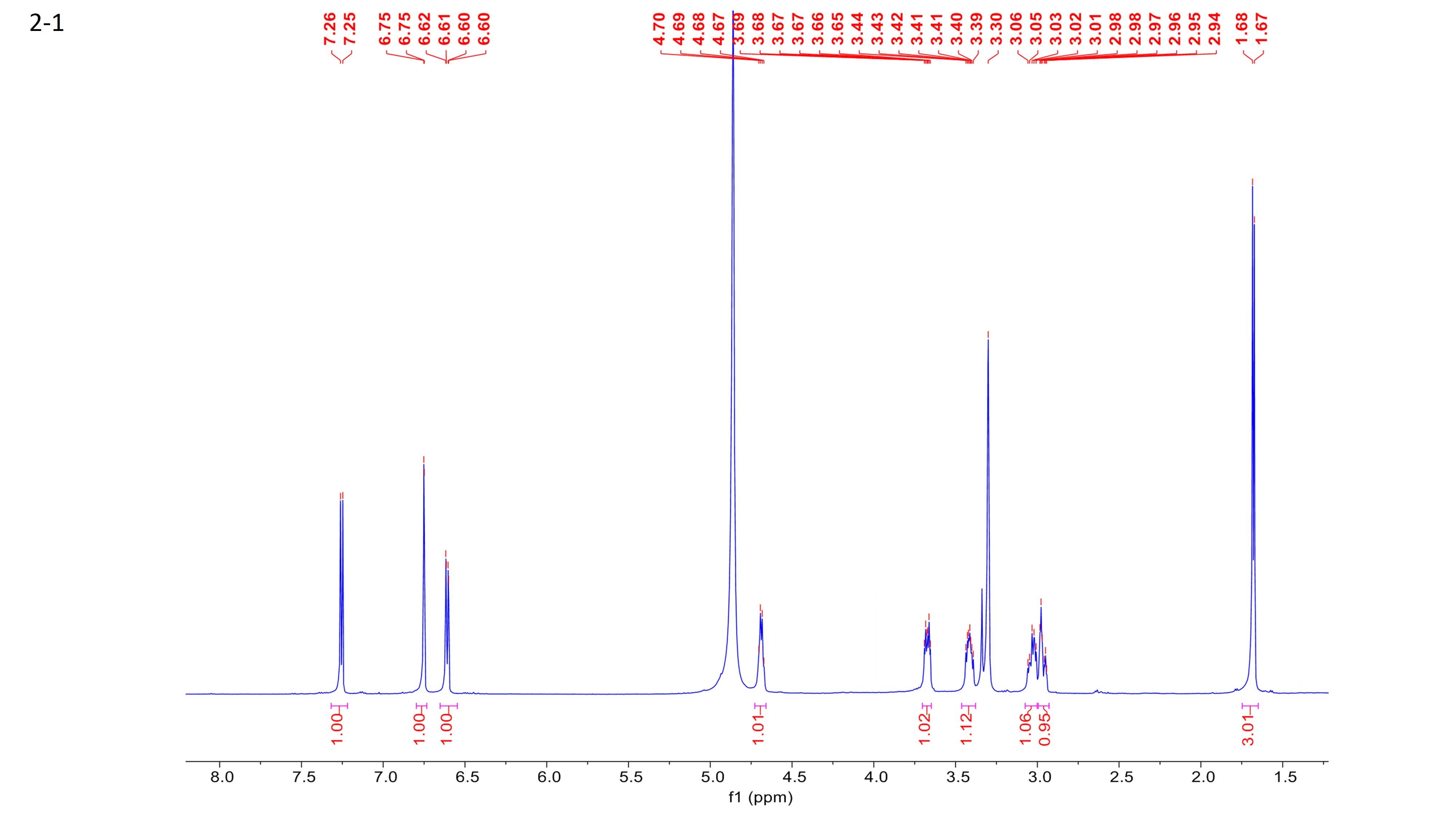

Supplement: Supplementary file 1 [file ijms-27-03757-s001.zip › Supplementary figure/SFr2-1/Figure S2 1H NMR Spectrum of Fr2-1(in DMSO-d6).jpg]

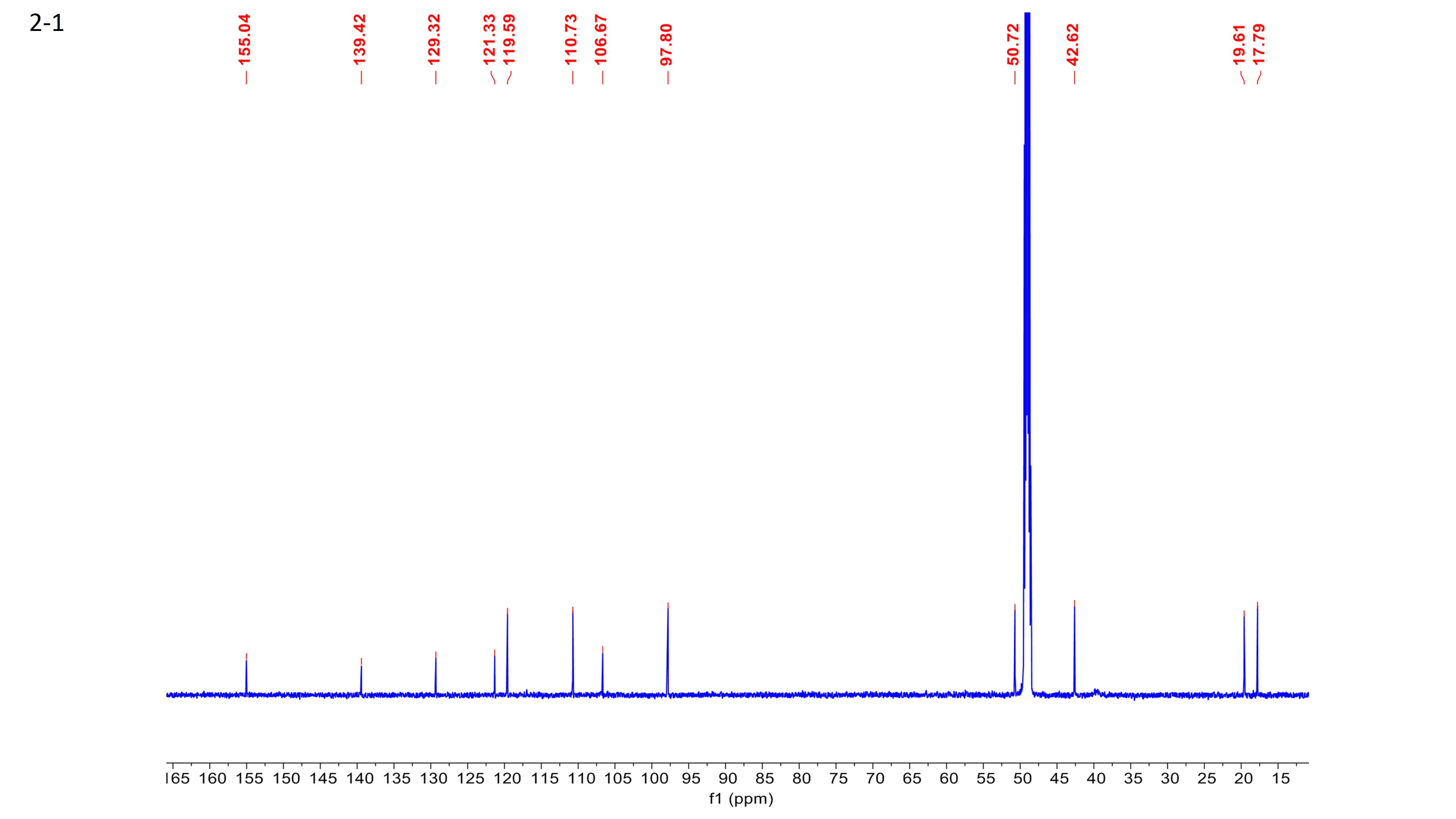

Supplement: Supplementary file 1 [file ijms-27-03757-s001.zip › Supplementary figure/SFr2-1/Figure S3 13C NMR Spectrum of Fr2-1(in DMSO-d6).jpg]

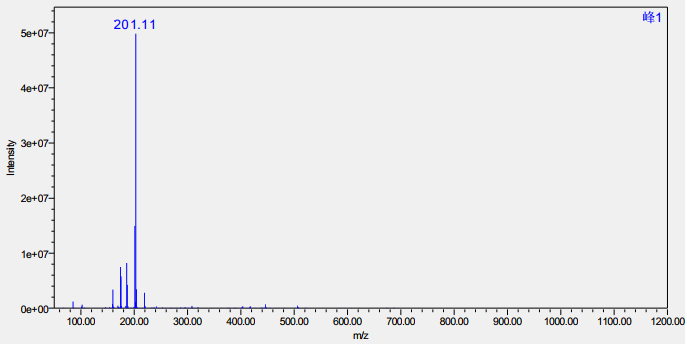

Supplement: Supplementary file 1 [file ijms-27-03757-s001.zip › Supplementary figure/SFr2-1/Figure S31 ESI mass spectrum of compound Fr2-1.png]

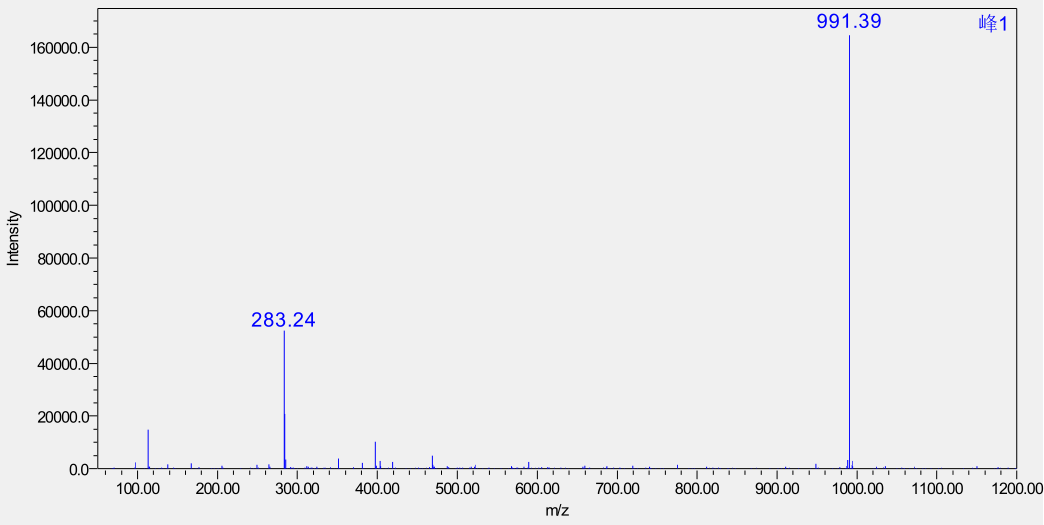

Supplement: Supplementary file 1 [file ijms-27-03757-s001.zip › Supplementary figure/SFr2-7/Figure S4 ESI mass spectrum of compound Fr2-7.jpg]

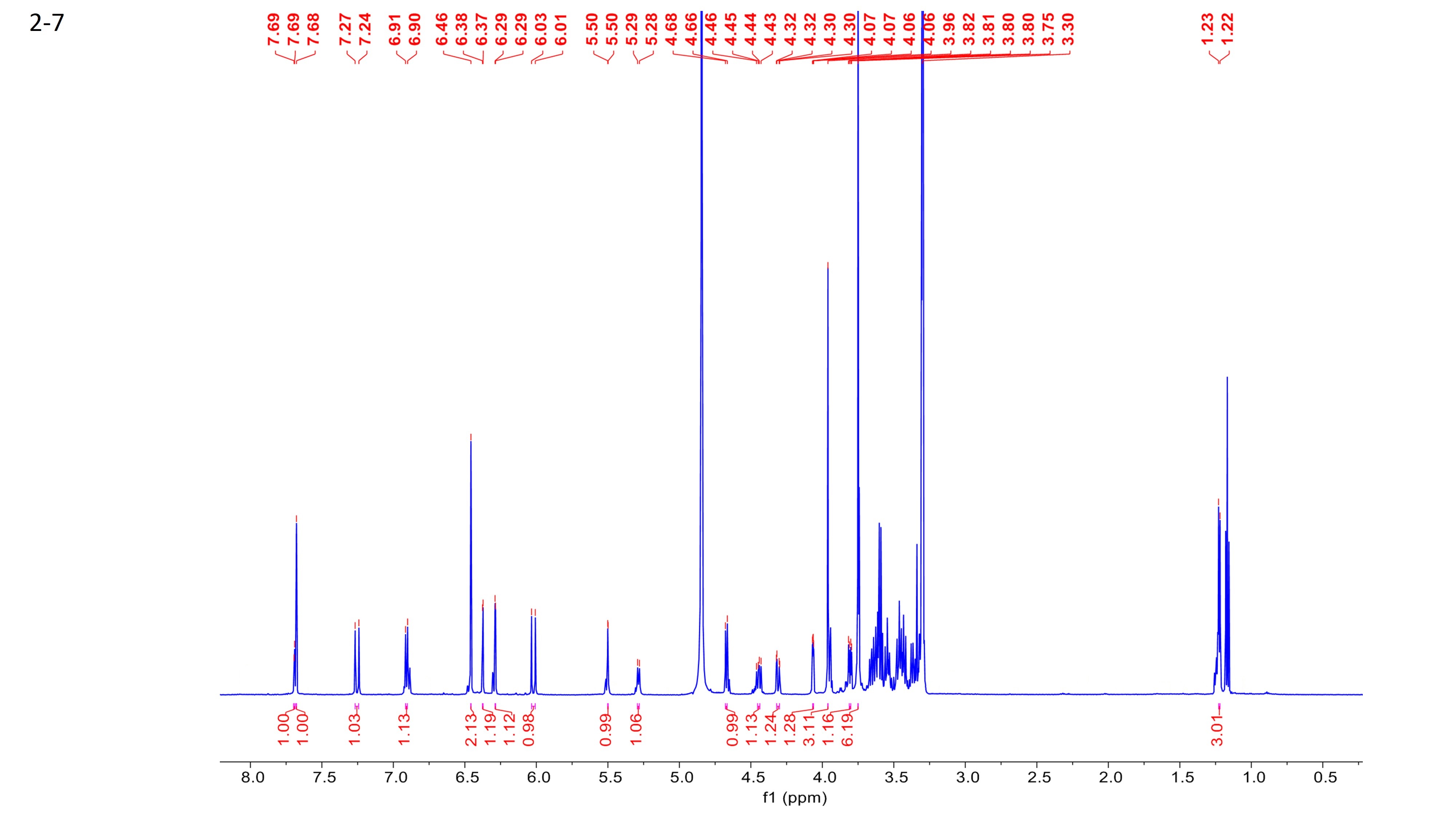

Supplement: Supplementary file 1 [file ijms-27-03757-s001.zip › Supplementary figure/SFr2-7/Figure S5 1H NMR Spectrum of Fr2-7(in DMSO-d6).jpg]

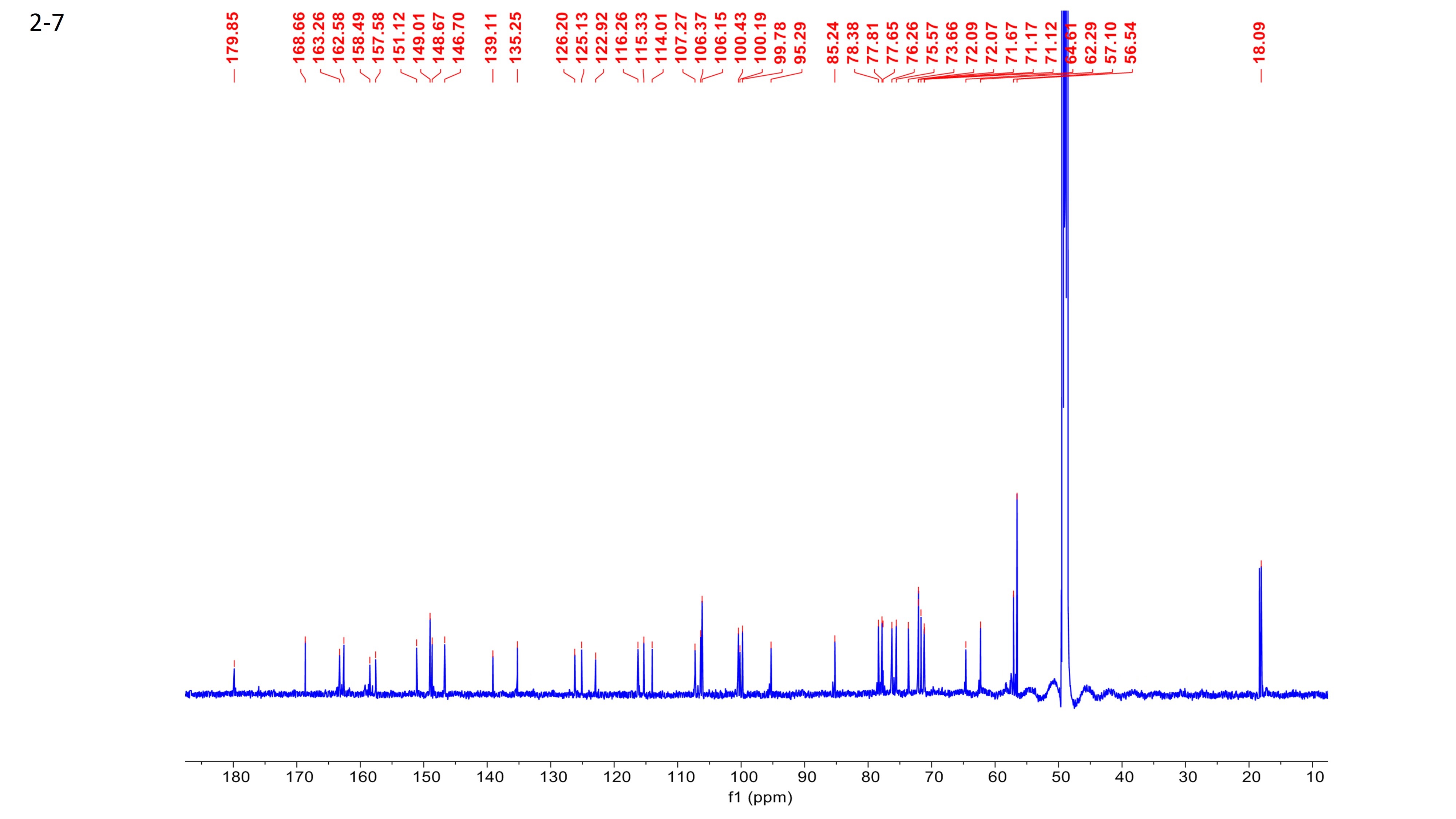

Supplement: Supplementary file 1 [file ijms-27-03757-s001.zip › Supplementary figure/SFr2-7/Figure S6 13C NMR Spectrum of Fr2-7(in DMSO-d6).jpg]
